# Extracellular Synthesis of Bioactive Silver Nanoparticles Using *Brevibacillus* sp. MAHUQ-41 and Their Potential Application Against Drug-Resistant Bacterial Pathogens *Listeria monocytogenes* and *Yersinia enterocolitica*

**DOI:** 10.3390/jfb16070241

**Published:** 2025-06-30

**Authors:** Md. Amdadul Huq

**Affiliations:** Department of Life Sciences, College of BioNano Technology, Gachon University, Seongnam 13120, Republic of Korea; amdadbge@gmail.com or mahuq@gachon.ac.kr

**Keywords:** *Brevibacillus* sp. MAHUQ-41, silver nanoparticles, extracellular synthesis, *Listeria monocytogenes*, *Yersinia enterocolitica*

## Abstract

The purpose of current study was the green synthesis of bioactive silver nanoparticles (AgNPs) using *Brevibacillus* sp. MAHUQ-41 and the exploration of their role in controlling drug-resistant bacterial pathogens *Listeria monocytogenes* and *Yersinia enterocolitica*. The culture supernatant of strain MAHUQ-41 was employed for a simple and eco-friendly synthesis of biofunctional silver nanoparticles (AgNPs). The resulting nanoparticles were analyzed using several techniques, including UV–Visible spectroscopy, XRD, FE-TEM, FTIR, and DLS. The UV–Vis spectral analysis of the AgNPs synthesized via *Brevibacillus* sp. MAHUQ-41 revealed a prominent absorption peak at 400 nm. FE-TEM results confirmed spherical-shaped 15–60 nm sized nanoparticles. XRD results indicated that the synthesized AgNPs were crystalline in nature. The FTIR spectrum determined various functional groups on the surface of synthesized nanoparticles. Potent antibacterial properties were observed in green-synthesized AgNPs against tested pathogens. The MIC value of extracellular synthesized AgNPs for both pathogenic bacteria was 6.2 µg/mL, and the MBCs were 25.0 µg/mL and 12.5 µg/mL for *L. monocytogenes* and *Y. enterocolitica,* respectively. Treatment by synthesized AgNPs resulted in morphological alterations and structural damages in both *L. monocytogenes* and *Y. enterocolitica*. These alterations can interfere with regular cellular activities, potentially resulting in cell death. This study is the first to report the antimicrobial properties of silver nanoparticles synthesized using *Brevibacillus* sp. MAHUQ-41. The findings obtained in the present study supported the role of *Brevibacillus* sp. MAHUQ-41-mediated synthesized AgNPs in controlling drug-resistant bacterial pathogens *L. monocytogenes* and *Y. enterocolitica*.

## 1. Introduction

Biomaterials are natural or artificial substances engineered to interface with biological systems for medical treatment or diagnostic use [1,2]. Bio-nanotechnology plays an important role in the creation of functional biomaterials by leveraging the distinctive characteristics of biological building blocks at the nanoscale. A key advantage of bio-nanotechnology is its ability to produce bioactive nanoparticles. Nanoparticles are functional nanomaterials and are the powerful therapeutic agents due to their unique characteristics, such as biocompatibility, non-toxicity, and multifunctional efficacy. Green-synthesized nanoparticles are attractive functional biomaterials because they offer several advantages over conventionally synthesized nanoparticles, including biocompatibility, improved properties, and environmentally friendly production methods [3,4,5]. This approach utilizes biological resources like plants or microorganisms, resulting in nanoparticles with specific biological functions and reduced toxicity. Nanomedicine has emerged as a promising agent for the safe and effective treatment of various deadly diseases, such as cancer and other infectious and non-infectious diseases. Nanoparticles and nanoconjugates have gained significant global attention due to their multifunctional applications in biomedical sectors [3,6]. Silver nanoparticles (AgNPs) are among the most extensively researched functional nanomaterials due to their diverse applications in biomedical fields, including antimicrobial activity, cancer treatment, targeted drug delivery, catalysis, and biomolecular sensing [7,8,9].

Nanoparticles can be synthesized using a range of approaches, such as chemical, physical, and biological methods [10,11,12]. Chemical and physical techniques are frequently employed to produce well-defined nanoparticles; however, they often require high energy input and involve harmful chemicals, resulting in toxic byproducts that limit their suitability [13,14]. The biosynthesis of nanoparticles is an emerging field of research due to their ease of use and environment friendly nature [15]. The green synthesis of nanoparticles has proven to be a safe, simple, non-hazardous, and biocompatible technique for developing nanomedicine using plants and microbes. Plant extracts contain various phytochemicals and microorganisms secreting numerous metabolites, which play a crucial role in the synthesis and stabilization of nanoparticles [4,16]. Green synthesis using microorganisms such as bacteria, yeast, actinomyces, and fungi has received increasing attention due to its simplicity and eco-friendliness [4,17,18,19]. Nanoparticle synthesis using bacterial biotransformation systems offers several benefits and has been recognized as an environmentally friendly approach [20,21]. Several bacterial species, including *Bacillus safensis* [22], *Escherichia coli* [23], *Sphingobium* sp. MAH-11 [9], *Acinetobacter baumannii* [24], *Terrabacter humi* [25], *Bacillus subtilis* [26], *Microbacterium resistens* [27], *Lactobacillus Plantarum* [28], *Pseudomonas deceptionensis* [29], and *Weissella oryzae* [30] have been studied for their role in the biosynthesis of AgNPs. In this study, we biologically synthesized novel AgNPs using *Brevibacillus* sp. MAHUQ-41 and evaluated their antimicrobial effects.

The lower effectiveness and side effects of available drugs as well as the emergence of drug-resistant microorganisms are serious concerns for public health globally. Consequently, there is a critical need to create new antimicrobial agents that are both safe and effective in fighting against drug-resistant pathogens. *Listeria monocytogenes* is a bacterial species responsible for listeriosis, an infection that can affect the brain, spinal cord membranes, and bloodstream of the host. This pathogen is commonly transmitted through the consumption of contaminated food, such as raw produce or unpasteurized dairy products. *L. monocytogenes* ranks as the third leading cause of fatal foodborne illness in humans, primarily affecting high-risk populations such as infants, the elderly, pregnant women, and individuals with weakened immune systems [31,32]. Similar to numerous other harmful pathogens, this bacterium has the potential to resist various antibiotics. There are many studies where the antimicrobial resistance in *L. monocytogenes* has been reported [33,34]. *Yersinia enterocolitica* is a Gram-negative, rod-shaped pathogen responsible for the zoonotic disease, yersiniosis. This infection typically presents with symptoms such as acute diarrhea, mesenteric lymphadenitis, terminal ileitis, and conditions mimicking appendicitis [35,36,37]. In many regions, infections caused by *Y. enterocolitica* pose a considerable public health challenge and lead to notable socioeconomic impacts [38]. Numerous studies have investigated antimicrobial resistance in *Y. enterocolitica* [38,39,40]. The development of safe and effective antibacterial agents is essential to control these pathogenic bacteria for protecting public health. Green-synthesized AgNPs appear to be a promising option for managing these bacterial pathogens. In the current study, a novel bacterial strain, *Brevibacillus* sp. MAHUQ-41, was isolated from the rhizosphere of the persimmon tree, and the culture supernatant of the strain MAHUQ-41 was utilized for the green, simple, and ecofriendly synthesis of bioactive AgNPs. Different instruments were used to analyze the properties of the synthesized AgNPs, including UV–Vis, TEM, EDX, XRD, DLS, and FTIR, and applied for the control of pathogenic *L. monocytogenes* and *Y. enterocolitica*. This is the first study on the green, simple synthesis of AgNPs using *Brevibacillus* sp. MAHUQ-41. It is also the first report on the antimicrobial application of *Brevibacillus* sp. MAHUQ-41-mediated synthesized AgNPs against human pathogens *L. monocytogenes* and *Y. enterocolitica*.

## 2. Materials and Methods

### 2.1. Chemicals and Materials

Silver nitrate (AgNO_3_) and culture media for bacteria were obtained from Sigma-Aldrich (St. Louis, MO, USA). The pathogenic bacteria *L. monocytogenes* [ATCC 19114] and *Y. enterocolitica* [ATCC 9610] were sourced from the American Type Culture Collection (ATCC, Manassas, VA, USA).

### 2.2. Isolation of AgNP-Producing Strain

The AgNP-producing strain *Brevibacillus* sp. MAHUQ-41 was isolated from the rhizosphere of a persimmon tree located in Anseong, South Korea, using the serial dilution method reported by Siddiqi et al. [41]. In brief, 1 g of each soil sample was combined with 9 mL of sterile 0.8% NaCl solution, shaken using a rotary shaker, and subsequently subjected to serial dilution. Each dilution (approximately 100 µL) was evenly spread over R2A agar plates. Following a 72 h incubation at 30 °C, individual colonies were selected based on their morphological characteristics. Each selected colony was then grown independently in 5 mL of R2A broth for 72 h at 30 °C. After incubation, 5 µL of a 1.5 M silver nitrate solution was introduced into the culture supernatant and further incubated in a shaking incubator at 35 °C for four days. Based on silver nitrate reduction efficacy, strain MAHUQ-41 was selected for further study. Strain MAHUQ-41 was deposited into the KACC.

### 2.3. 16S rRNA Gene Sequencing and Phylogenetic Analysis

Strain MAHUQ-41 was identified at the molecular level through 16S rRNA gene sequencing. Genomic DNA was extracted using a commercially available DNA isolation kit (Solgent, Republic of Korea). The 16S rRNA gene amplification was conducted using primers 27F and 1492R, followed by sequencing at Solgent Co., Ltd., in Daejeon, Republic of Korea. Identification of strain MAHUQ-41 was achieved by comparing the obtained 16S rRNA sequence with entries in the GenBank database using the BLASTn tool. To determine the phylogenetic relationship of MAHUQ-41, a phylogenetic tree was generated employing the neighbor-joining approach via MEGA6 software [42,43].

### 2.4. Cultural, Physiological, and Biochemical Characterization of Strain MAHUQ-41

To evaluate its growth characteristics, strain MAHUQ-41 was incubated at 30 °C for three days on different agar media, including trypticase soy agar, nutrient agar, Luria-Bertani agar, and R2A agar [44]. To determine the optimal growth temperature, the strain was cultured on R2A agar and incubated at temperatures ranging from 5 °C to 50 °C in 5 °C intervals [45]. For pH optimization, strain MAHUQ-41 was grown in R2A broth adjusted to different pH levels [46]. Transmission electron microscopy was used to observe the cell’s shape and size. Using API 20NE and API ZYM kits (bioMérieux), the physiological and biochemical properties of strain MAHUQ-41 were evaluated following the provided protocols.

### 2.5. Biosynthesis of AgNPs Using Strain MAHUQ-41

The culture supernatant of the potent strain MAHUQ-41 was employed to synthesize novel AgNPs. The isolated strain MAHUQ-41 was cultured in 100 mL R2A broth for 3 days at 30 °C under shaking at 180 rpm. Afterward, the supernatant was separated by centrifuging at 8000 rpm for 10 min. AgNPs were produced by combining the culture supernatant with silver nitrate solution, achieving a final concentration of 1.5 mM. The reaction was carried out in the dark at 35 °C with continuous shaking at 180 rpm in an orbital shaker for four days. The synthesis of bioactive AgNPs was regularly observed, and the completion of synthesis was confirmed using UV–Visible spectroscopy. The resulting AgNPs were harvested by centrifugation at 14,000 rpm for 20 min, followed by washing with deionized water. Afterward, the nanoparticles were air-dried and used for further characterization and antibacterial testing.

### 2.6. Characterization of Green-Synthesized AgNPs

The synthesized AgNPs were analyzed using a UV–Visible spectrophotometer (Optizen POP, Mecasys) to study their kinetic properties. Spectral scanning of the biosynthesized AgNPs was performed over the wavelength range of 300–800 nm, and the absorption data were recorded and graphed. Morphology, particle size, purity, elemental composition, distribution, and selected area electron diffraction (SAED) patterns of the green-synthesized AgNPs were examined using a field emission transmission electron microscope (FE-TEM) equipped with an energy-dispersive X-ray (EDX) detector. For TEM imaging, a drop of AgNP suspension was placed on a carbon-coated copper grid, air-dried, and then examined with a FE-TEM (TEM-2100F, Joel, Tokyo, Japan) operated at 200 kV. The crystalline structure of AgNPs was further analyzed through X-ray diffraction (XRD) using a D8 Advance diffractometer (Bruker, Germany). The diffractometer operates using CuKα radiation at 40 mA and 40 kV over the range of 30° to 80° (2θ). The surface chemistry of the biosynthesized AgNPs was investigated using FTIR (Fourier transform-infrared) spectroscopy (Spectrum One System, Perkin-Elmer, Waltham, MA, USA) with a resolution of 4 cm^−1^ in the range of 500–4000 cm^−1^. For FTIR analysis, the air-dried powder form of synthesized AgNPs was used. The hydrodynamic diameters and polydispersity index (PDI) of the AgNPs were measured using Dynamic Light Scattering (DLS) with a Malvern Zetasizer Nano ZS90 (Malvern Instruments, Worcestershire, UK). Deionized water, with a viscosity of 0.8878 and a refractive index of 1.3328, was used as the dispersing medium, and measurements were performed at a controlled temperature of 25.0 °C.

### 2.7. Antimicrobial Activity

The antimicrobial activity of the biosynthesized AgNPs was assessed against *L. monocytogenes* and *Y. enterocolitica* using the disc diffusion technique [47,48]. In brief, both bacterial strains were grown overnight in Mueller–Hinton (MH) broth. Each culture (100 µL) was evenly distributed across MH agar plates. A suspension was prepared by dissolving 1 mg of the synthesized AgNPs in 2 mL of sterile distilled water. Sterile paper discs were impregnated with either 50 µL or 100 µL of this AgNP solution and placed on the inoculated agar surfaces. As controls, six antibiotics—penicillin G (10 µg/disc), novobiocin (30 µg/disc), oleandomycin (15 µg/disc), erythromycin (15 µg/disc), vancomycin (30 µg/disc), and lincomycin (15 µg/disc)—were tested against both bacterial strains. Following a 24 h incubation at 37 °C, the diameter of the inhibition zones (ZOI) on the agar plates was recorded in millimeters [48,49].

### 2.8. MIC and MBC

The minimum inhibitory concentration (MIC) and minimum bactericidal concentration (MBC) of AgNPs were assessed against the pathogenic strains *L. monocytogenes* and *Y. enterocolitica*. Using 96-well ELISA plates, the MIC was determined through the broth microdilution method. Both strains were cultured in MH broth overnight. A 100 µL aliquot of bacterial suspension (approximately 1 × 10^6^ CFU/mL) was dispensed into each well of a 96-well ELISA plate, followed by the addition of 100 µL of AgNP solutions at concentrations ranging from 1.56 to 100 µg/mL. The minimum concentration of AgNPs that prevented microbial growth was determined as the MIC. The MBC was determined by transferring 10 µL from each well of the 96-well plate onto MH agar plates, followed by incubation at 37 °C for 24 h. No visible bacterial growth at the lowest AgNP concentration was noted in the MBC. The experimental procedures were conducted in accordance with previously published protocols [48,49].

### 2.9. Morphological Evaluation

To investigate the antibacterial action of synthesized AgNPs against *L. monocytogenes* and *Y. enterocolitica*, both bacterial strains (approximately 1 × 10^7^ CFU/mL) were incubated at 37 °C in the presence and absence of nanoparticles at the MBC concentration. Following incubation, cells were pelleted by centrifuging at 8000 rpm for 5 min. After washing with buffer, the cells were fixed with 2.5% glutaraldehyde followed by 1% osmium tetroxide. The fixed samples were subsequently dehydrated using a graded ethanol series and dried in a desiccator. Morphological and structural changes in *L. monocytogenes* and *Y. enterocolitica* were examined via FE-SEM (field emission scanning electron microscopy) [48,50].

## 3. Results and Discussion

### 3.1. Molecular Identification of AgNP-Producing Bacteria

**Figure 1:**
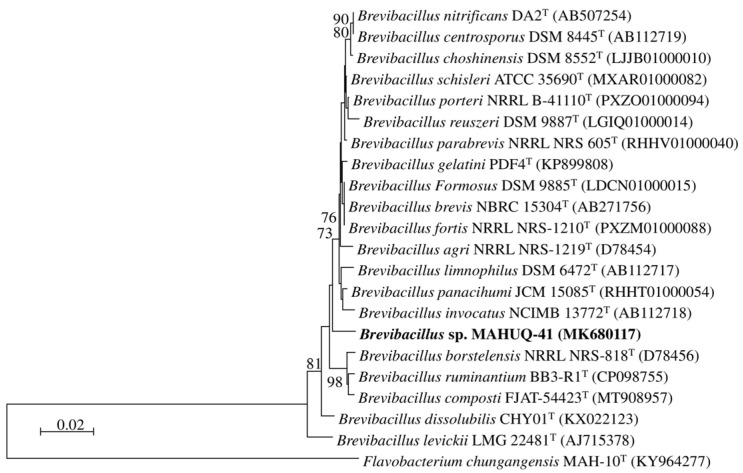
A neighbor-joining (NJ) phylogenetic tree was constructed using 16S rRNA gene sequences to illustrate the evolutionary relationships between *Brevibacillus* sp. MAHUQ-41 and closely related type strains.

Strain MAHUQ-41’s 16S rRNA gene sequence, measuring 1210 bp, was submitted to the GenBank/EMBL/DDBJ database under accession number MK680117. Comparisons based on 16S rRNA gene sequences revealed that strain MAHUQ-41 was closely related to *Brevibacillus choshinensis* DSM 8552^T^, *Brevibacillus formosus* DSM 9885^T^, and *Brevibacillus brevis* NBRC 15304^T^ a with sequence similarity of 98.7%. Phylogenetic analysis using 16S rRNA gene sequence also showed that strain MAHUQ-41 clusters with the members of the genus *Brevibacillus* (Figure 1). Strain MAHUQ-41 was deposited into the KACC (Deposition number KACC 21235).

### 3.2. Cultural, Physiological, and Biochemical Characterization of Strain MAHUQ-41

**Figure 2:**
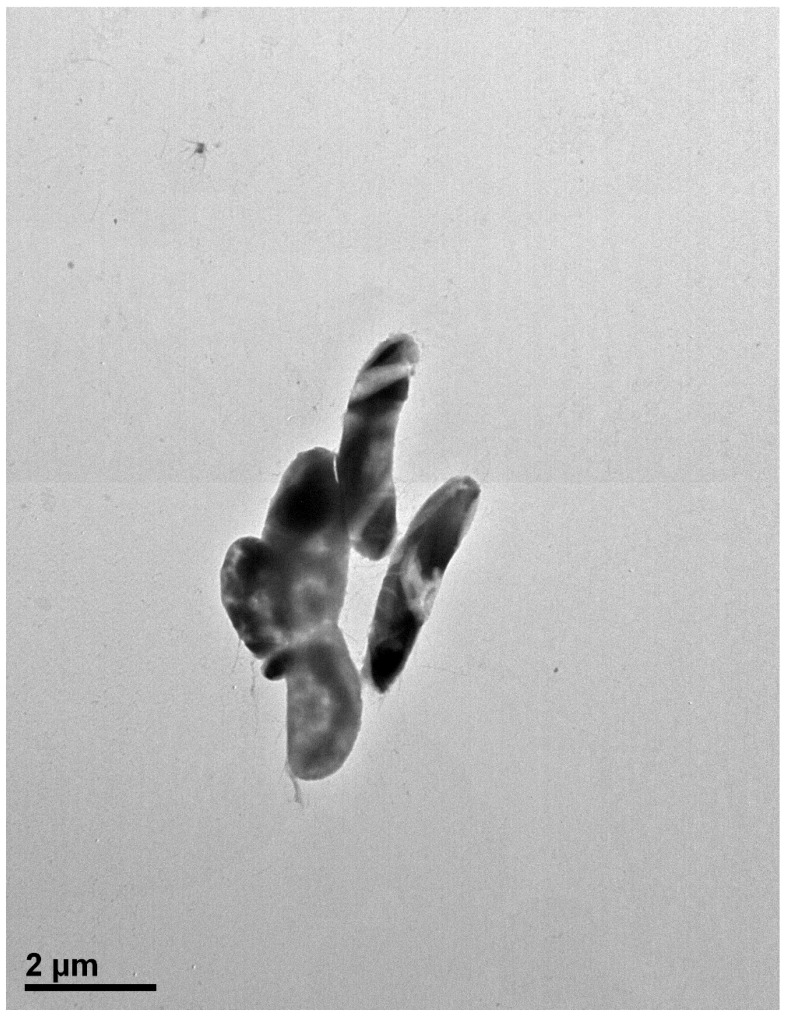
Transmission electron microscopy image of *Brevibacillus* sp. MAHUQ-41 cells following negative staining with uranyl acetate. Scale bar represents 2.0 μm.

The cells of strain MAHUQ-41 are rod shaped (Figure 2). Colonies grow well on R2A agar medium with an optimum temperature of 30 °C and pH of 7.0–7.5. Strain MAHUQ-41 shows positive activity for the hydrolysis of gelatin. The strain MAHUQ-41 shows the following enzyme activities: acid phosphatase, alkaline phosphatase, napthol-AS-BI-phosphohydrolase, esterase lipase (C8), and leucine arylamidase.

### 3.3. Green Synthesis of AgNPs

**Figure 3:**
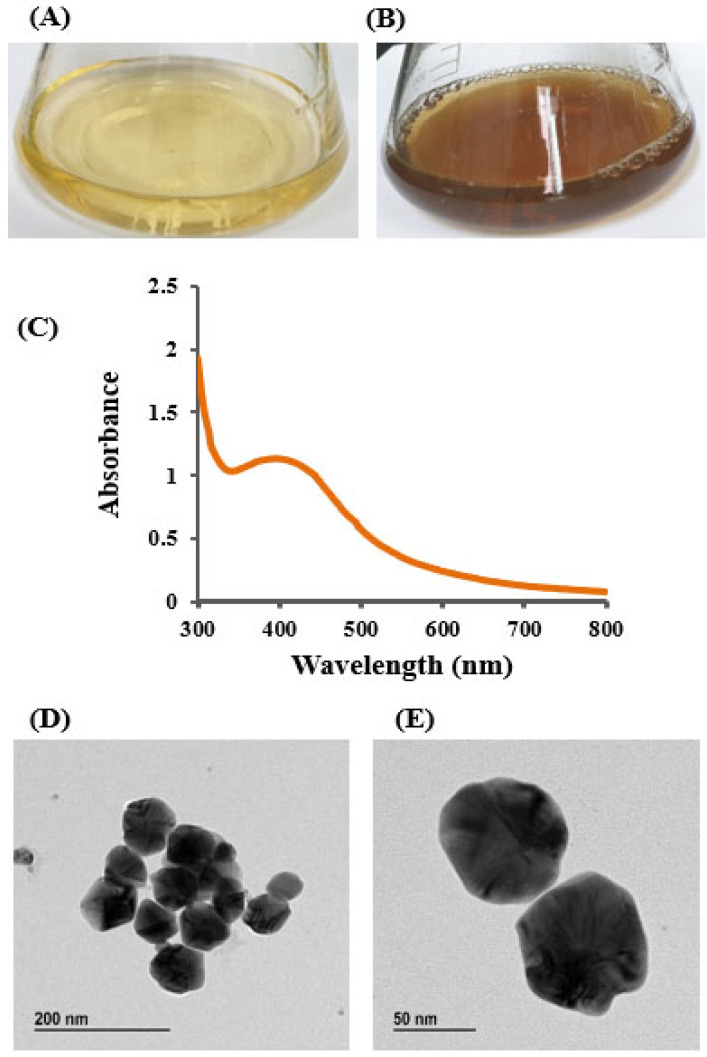
R2A broth with AgNO_3_ as control (**A**), biosynthesized AgNPs (**B**), UV–Vis spectra (**C**), and FE-TEM images of AgNPs (**D**,**E**) synthesized by *Brevibacillus* sp. MAHUQ-41.

The bioactive AgNPs were synthesized using the culture supernatant of isolated strain *Brevibacillus* sp. MAHUQ-41. Strain MAHUQ-41 showed strong ability in the reduction of silver ion, leading to the synthesis of AgNPs. The synthesis of AgNPs was visually confirmed by a distinct color change in the reaction mixture, shifting from pale yellow to brown, a transformation attributed to the surface plasmon resonance (SPR) typically observed in green-synthesized AgNPs (Figure 3A,B) [48,51]. No noticeable color change occurred in the control sample containing R2A broth with AgNO_3_ (Figure 3A). The color transition occurred within four days of incubation (Figure 3B), serving as an initial visual indicator of successful AgNP formation mediated by *Brevibacillus* sp. MAHUQ-41 [48,52]. Similar findings were reported by Wang et al. [27], who observed a deep brown coloration in the reaction mixture containing the culture supernatant of *Bacillus sonorensis* and AgNO_3_. Hamouda et al. further documented a comparable brown color change, confirming AgNP formation [52]. In the present work, an extracellular synthesis method was employed to facilitate a straightforward and efficient production of AgNPs using the culture supernatant of *Brevibacillus* sp. MAHUQ-41. Although the exact mechanism behind AgNP synthesis remains unclear, several studies suggest the involvement of microbial enzymes and biomolecules such as proteins, pigments, flavonoids, enzymes, and amino acids in nanoparticle formation [4,53]. Microbial culture supernatants and biomass possess various bioactive substances, such as enzymes, proteins, amino acids, and other biomolecules, which function as reducing, capping, and stabilizing agents in the nanoparticle synthesis process [4,9]. In particular, microbial reductase enzymes are believed to play a pivotal role in reducing metal ions to their nanoparticle form [4,9,54,55].

### 3.4. Characterization of Synthesized AgNPs

The appearance of a brown coloration in the reaction mixture served as an initial visual indication of AgNP synthesis. This observation was further validated using UV–Vis spectroscopy, which is commonly employed to monitor the surface plasmon resonance (SPR) associated with nanoparticle formation. The UV–Vis analysis confirmed the successful synthesis of AgNPs by the culture supernatant of *Brevibacillus* sp. MAHUQ-41, as evidenced by a characteristic broad absorption peak around 400 nm (Figure 3C), typical of AgNPs. This coincided with the plasmon resonance characteristic of AgNPs [21,52]. Hamouda et al. found the absorption peak of cyanobacterium *Oscillatoria limnetica*-mediated synthesized AgNPs at 426 nm [52]. An absorption peak at 413 nm was observed in AgNPs synthesized from the culture supernatant of *Terrabacter humi* [25]. The distinct UV–Vis absorption peak observed between 400 and 500 nm during the formation of silver nanoparticles (AgNPs) arises from surface plasmon resonance (SPR). This effect results from the collective oscillation of conduction electrons on the nanoparticle surface in response to incident light. The exact position of this resonance peak is influenced by factors such as the nanoparticle’s size, shape, and surrounding dielectric medium [56,57]. TEM was used to analyze the morphology, size, and distribution of AgNPs synthesized by *Brevibacillus* sp. MAHUQ-41. As shown in Figure 3D,E, the AgNPs were predominantly spherical, with particle sizes ranging from 15 to 60 nm. The TEM images also indicated a uniform distribution of nanoparticles, with no visible signs of aggregation, suggesting that the biosynthesized AgNPs possess good stability. These findings are in agreement with those of Singh et al. [58], who reported AgNP sizes between 10 and 40 nm when synthesized using the culture supernatant of *Pseudomonas* sp. THG-LS1.4. Similarly, *Paenarthrobacter nicotinovorans* MAHUQ-43 was shown to produce spherical AgNPs with sizes ranging from 13 to 27 nm [21]. Differences in average particle size among studies may be attributed to variations in the metabolites secreted by different biological sources used as reducing, capping, and stabilizing agents.

**Figure 4:**
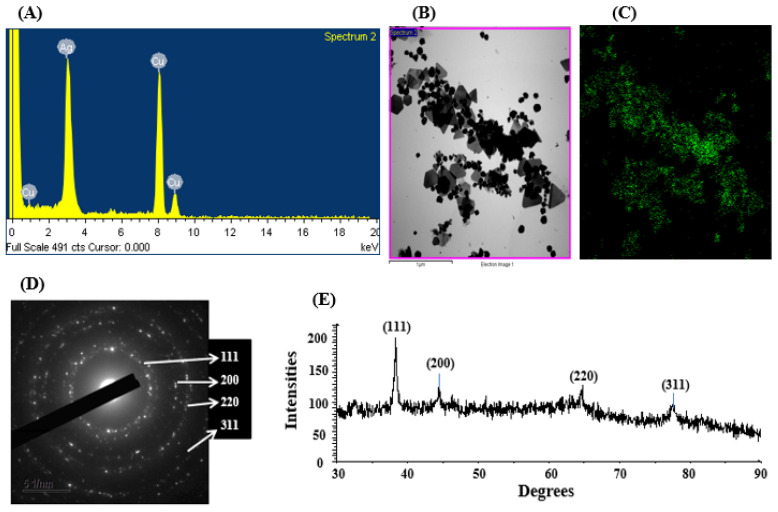
EDX pattern of synthesized AgNPs (**A**), TEM image utilized for mapping (**B**), silver distribution in elemental mapping (**C**), SAED pattern (**D**), and X-ray diffraction pattern (**E**) of *Brevibacillus* sp. MAHUQ-41-mediated green-synthesized AgNPs.

Elemental compositions and their distribution were examined by an EDX detector. The data revealed the main element in the green-synthesized nanomaterial was silver (Figure 4A–C). The EDX spectrum of the synthesized nanoparticles exhibited a prominent peak at 3 keV, characteristic of silver, confirming the presence of AgNPs (Figure 4A). Additional elemental signals detected in the spectrum were attributed to the TEM grid used during FE-TEM imaging [58]. The elemental composition obtained from the EDX analysis is presented in Table 1. Similar EDX profiles were observed in AgNPs produced by the culture supernatant of *Pseudomonas* sp. THG-LS1.4 [58] and *Massilia* sp. MAHUQ-52 [59]. SAED patterns further confirmed the crystalline nature of the AgNPs, showing distinct rings indicative of metallic and crystalline features (Figure 4D). The crystalline nature of the green-synthesized AgNPs was also verified by XRD analysis, with results displayed in Figure 4E. The XRD pattern displayed four distinct peaks at 2θ values of 38.51°, 44.38°, 64.69°, and 77.86°, corresponding to the (111), (200), (220), and (311) planes, respectively, which are characteristic of metallic silver (Figure 4E) [58]. Similar results were reported in earlier studies, where *Pseudomonas* sp. THG-LS1.4-mediated and *Paenarthrobacter nicotinovorans* MAHUQ-43-derived AgNPs exhibited similar XRD patterns [21,58].

**Figure 5:**
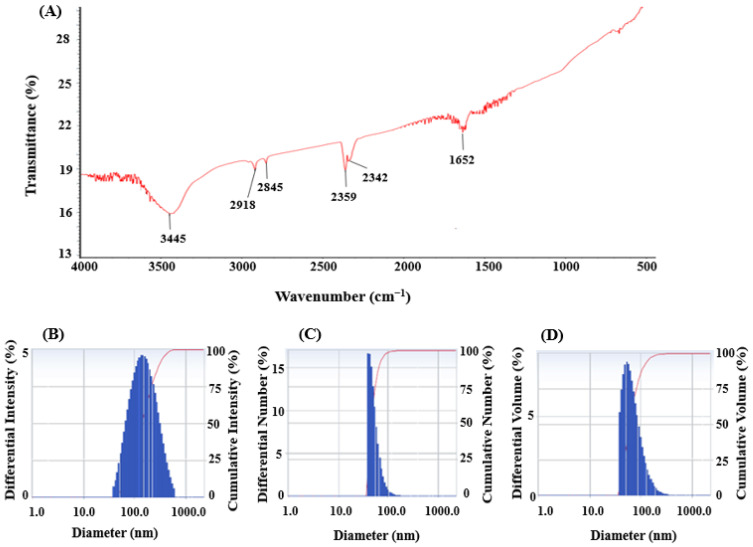
FT-IR spectra (**A**) and particle size distribution of green-synthesized AgNPs mediated by *Brevibacillus* sp. MAHUQ-41, presented by intensity (**B**), number (**C**), and volume (**D**).

FT-IR analysis was conducted to detect the presence of functional groups involved in the reduction and stabilization of silver ions during the green synthesis of AgNPs mediated by *Brevibacillus* sp. MAHUQ-41. The FT-IR spectrum displayed several prominent transmission peaks at 3445, 2918, 2845, 2359, 2342, and 1652 cm^−1^ (Figure 5A). The strong absorption band at 3445 cm^−1^ is indicative of O–H stretching vibrations from alcohols and/or N–H stretching from amine groups. The bands found at 2918 and 2845 cm^−1^ in *Brevibacillus* sp. MAHUQ-41-mediated green-synthesized AgNPs are due to the C-H bonds. The stretching peaks at 2359 and 2342 cm^−1^ corresponded to the O=C=O (carbonyl bond group) stretching. The peak observed at 1652 cm^−1^ corresponded to the C=O, –C=C, or N–H functional group. The presence of multiple functional groups in the FTIR spectrum suggests that a variety of biomolecules may play a role in both the reduction of silver ions and the stabilization of the synthesized AgNPs. These findings align with earlier research indicating that biomolecular components in microbial cell extracts contribute significantly to nanoparticle synthesis and stability [9,27,52]. DLS analysis revealed that the *Brevibacillus* sp. MAHUQ-41-mediated AgNPs had an average hydrodynamic diameter of 129.5 nm, with a polydispersity index (PDI) of 0.235, indicating that the green-synthesized AgNPs had a polydisperse standard (Figure 5B–D). The DLS analysis revealed the larger size of AgNPs than the actual size identified by FE-TEM analysis could be due to the presence of water molecules on the surface of AgNPs.

### 3.5. Antibacterial Activity

**Figure 6:**
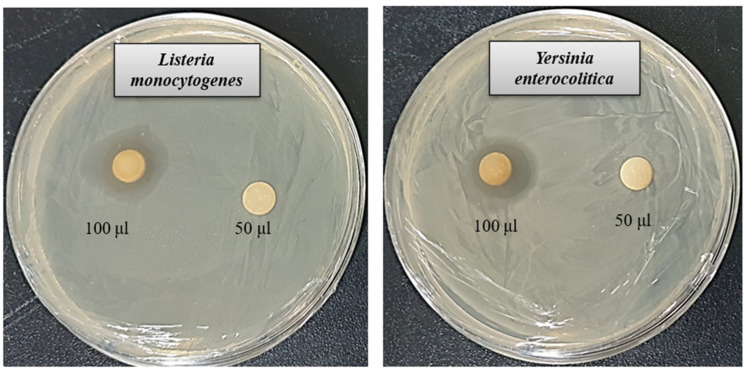
Antibacterial activity of the synthesized AgNPs. Inhibition zones observed against *L. monocytogenes* and *Y. enterocolitica* using 50 µL and 100 µL of 500 ppm concentrations.

The rise of multidrug-resistant microbes presents a significant challenge to global health. Due to the limited effectiveness of existing antibiotics, resistant strains have become more prevalent, highlighting the pressing demand for novel and potent antimicrobial solutions. AgNPs synthesized through biological methods have shown promise as powerful antimicrobial agents capable of targeting harmful pathogens. This study assessed the antibacterial properties of AgNPs produced using *Brevibacillus* sp. MAHUQ-41 against the antibiotic-resistant bacteria *L. monocytogenes* and *Y. enterocolitica*. The biologically derived AgNPs demonstrated marked antibacterial activity against both strains. As shown in Figure 6, a distinct zone of inhibition (ZOI) was observed. The ZOI diameters for the AgNPs synthesized by *Brevibacillus* sp. MAHUQ-41 were measured at 19.1 ± 1.5 mm for *L. monocytogenes* and 18.9 ± 1.3 mm for *Y. enterocolitica* when exposed to a 100 µL solution at a concentration of 500 ppm (Table 2). These results indicate that biosynthesized AgNPs have the potential to effectively suppress these pathogenic bacteria.

**Figure 7:**
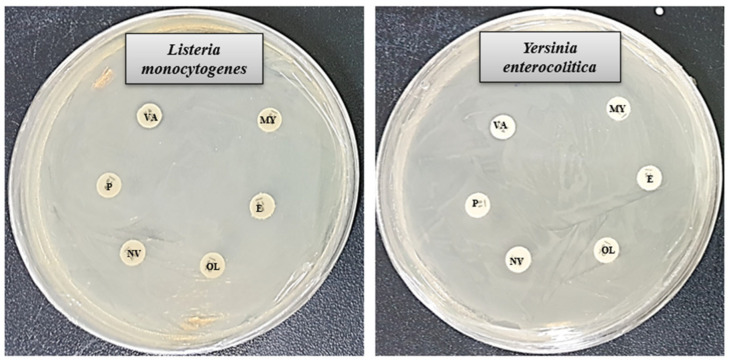
ZOI of tested antibiotics against *L. monocytogenes* and *Y. enterocolitica*. Abbreviation: P (penicillin G), NV (novobiocin), E (erythromycin), VA (vancomycin), OL (oleandomycin), and MY (lincomycin).

In this investigation, the antibacterial performance of six commercially available antibiotics (penicillin G, novobiocin, erythromycin, vancomycin, oleandomycin, and lincomycin) was evaluated and compared with that of biosynthesized AgNPs against *L. monocytogenes* and *Y. enterocolitica*. The findings revealed that most of the tested antibiotics showed little to no effectiveness against either bacterial strain. Notably, only vancomycin displayed limited activity against *L. monocytogenes*, while novobiocin exhibited mild effects on *Y. enterocolitica*. The diameter of the ZOI of vancomycin against *L. monocytogenes* was 8.9 ± 1.2, and the diameter of the ZOI of novobiocin against *Y. enterocolitica* was 10.2 ± 1.0 (Table 3). These results are consistent with earlier research highlighting the potent antimicrobial capabilities of biosynthesized AgNPs [58,59]. The observed antibacterial efficacy of the synthesized AgNPs suggests their promise as alternative therapeutic agents for combating multidrug-resistant pathogens. A detailed presentation of the results is shown in Figure 7 and Table 3.

### 3.6. MIC and MBC

**Figure 8:**
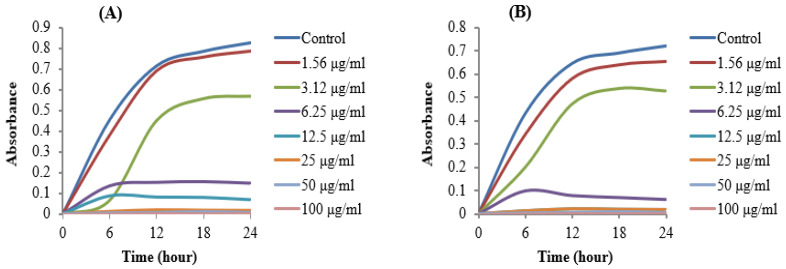
MICs of biosynthesized AgNPs against *L. monocytogenes* (**A**) and *Y. enterocolitica* (**B**).

The MIC of AgNPs synthesized by *Brevibacillus* sp. MAHUQ-41 was determined using the standard microdilution method. Various concentrations of the biosynthesized AgNPs (1.56, 3.12, 6.25, 12.5, 25, 50, and 100 µg/mL) were tested against *L. monocytogenes* and *Y. enterocolitica*. The results showed that both bacterial strains were inhibited at a MIC of 6.25 µg/mL. This indicates that the AgNPs synthesized by *Brevibacillus* sp. MAHUQ-41 are highly effective in suppressing the growth of these pathogens (Figure 8A,B). Notably, the MIC values observed in this study were considerably lower than those reported for other antimicrobial agents used against *L. monocytogenes* and *Y. enterocolitica* [60,61,62], underscoring the superior antimicrobial potential of the biosynthesized AgNPs.

**Figure 9:**
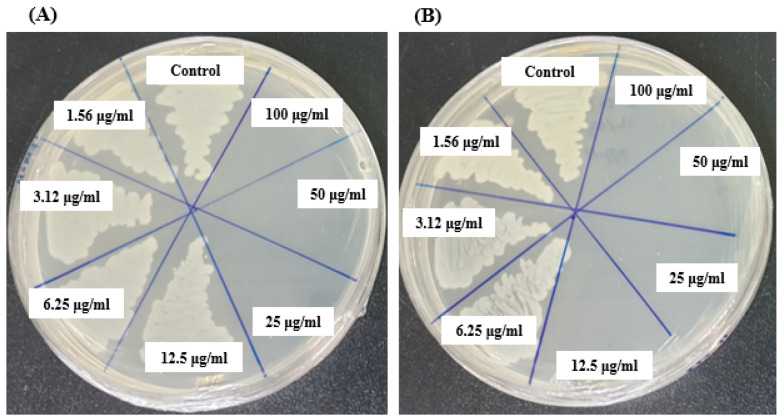
MBCs of biosynthesized AgNPs against *L. monocytogenes* (**A**) and *Y. enterocolitica* (**B**).

The MBC of AgNPs synthesized by *Brevibacillus* sp. MAHUQ-41 were determined to be 25 µg/mL for *L. monocytogenes* and 12.5 µg/mL for *Y. enterocolitica* (Figure 9A,B). These findings confirm the strong bactericidal efficacy of the *Brevibacillus* sp. MAHUQ-41-mediated synthesized AgNPs in suppressing the growth of both pathogenic *L. monocytogenes* and *Y. enterocolitica.*

### 3.7. Morphological Evaluation

**Figure 10:**
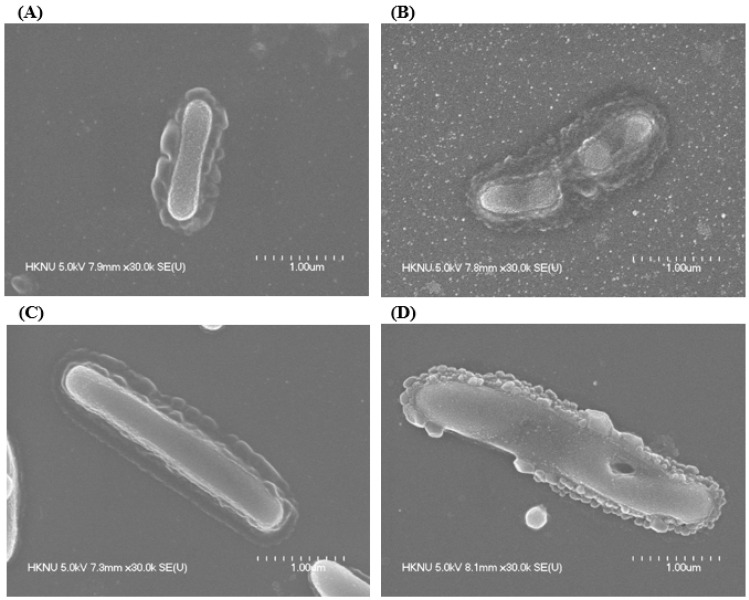
Normal *L. monocytogenes* (**A**), AgNP-treated *L. monocytogenes* (**B**), normal *Y. enterocolitica* (**C**), AgNP-treated *Y. enterocolitica* (**D**).

FE-SEM was employed to examine the morphological alterations in *L. monocytogenes* and *Y. enterocolitica* following treatment with AgNPs synthesized by *Brevibacillus* sp. MAHUQ-41 (Figure 10). Untreated *L. monocytogenes* cells maintained their typical rod-like shape and displayed smooth, intact surfaces (Figure 10A). In contrast, cells exposed to a 1 × MBC concentration of the biosynthesized AgNPs exhibited significant surface irregularities, structural damage, and deformation, resulting in complete membrane collapse (Figure 10B). A similar pattern was observed in *Y. enterocolitica*. While the untreated cells appeared structurally intact, with their characteristic rod shape (Figure 10C), those treated with AgNPs showed notable surface damage, deformation, and loss of structural integrity (Figure 10D). These morphological disruptions suggest that the AgNPs interfere with cellular architecture and may lead to the loss of vital cell functions, ultimately causing bacterial cell death. Numerous antimicrobial actions of AgNPs were identified. Among the most recognized are the disruption of microbial cell membranes, the production of reactive oxygen species (ROS), and interactions with cellular DNA and proteins, all of which contribute to microbial cell death [4,63]. AgNPs compromise the structure of bacterial cell walls and membranes, increasing membrane permeability, which leads to the leakage of intracellular contents and ultimately causes cell death. They also disrupt the respiratory chain by binding to sulfhydryl groups, triggering lipid peroxidation and oxidative damage to DNA and proteins, resulting in cell destruction [4,64,65]. Furthermore, AgNPs attach to sulfur and phosphorus groups in DNA, causing DNA aggregation and damage, which interferes with transcription and translation processes. These nanoparticles can also promote the dephosphorylation of phosphotyrosine residues, thereby disturbing cellular signal transduction pathways and leading to cell death. AgNPs may release silver ions (Ag^+^) from their surfaces. These ions play a critical antimicrobial role by interacting with bacterial cell wall and membrane structures, representing a key mechanism in AgNP-induced toxicity [4,66,67].

## 4. Conclusions

This study is the first to report the green and straightforward synthesis of AgNPs utilizing *Brevibacillus* sp. MAHUQ-41. It also marks the initial application of these biosynthesized AgNPs in targeting the pathogenic bacteria *L. monocytogenes* and *Y. enterocolitica*. The culture supernatant of strain MAHUQ-41 functioned efficiently for the eco-friendly synthesis of AgNPs. The extracellular methodology was used for nanoparticle synthesis, which is a simple, fast, reproducible, and eco-friendly approach. The AgNPs produced through green synthesis were analyzed using techniques such as UV–Vis spectroscopy, FE-TEM, XRD, DLS, and FTIR. The UV–Vis spectrum of *Brevibacillus* sp. MAHUQ-41-mediated green-synthesized AgNPs showed a strong peak at 400 nm. FE-TEM results confirmed a spherical shape 15–60 nm in size. XRD analysis confirmed the crystalline nature of the synthesized AgNPs. FTIR analysis detected multiple biomolecules on the AgNPs’ surface, indicating their biological origin. The AgNPs synthesized using *Brevibacillus* sp. MAHUQ-41 exhibited strong antimicrobial efficacy against drug-resistant pathogenic *L. monocytogenes* and *Y. enterocolitica*. The nanoparticles created distinct inhibition zones measuring 19.1 ± 1.5 mm for *L. monocytogenes* and 18.9 ± 1.3 mm for *Y. enterocolitica*. The MIC for both bacterial strains was found to be 6.2 µg/mL, while the MBCs were 25.0 µg/mL for *L. monocytogenes* and 12.5 µg/mL for *Y. enterocolitica*. FE-SEM revealed significant morphological and structural disruptions in *L. monocytogenes* and *Y. enterocolitica* cells following treatment with the synthesized AgNPs. These observed alterations are likely to impair essential cellular functions in these multi-drug-resistant *L. monocytogenes* and *Y. enterocolitica*, ultimately resulting in cell death. The findings of this study suggest that the culture supernatant of *Brevibacillus* sp. MAHUQ-41 offers a straightforward and eco-friendly approach for synthesizing AgNPs, which demonstrate strong potential as effective antimicrobial agents against drug-resistant human pathogens such as *L. monocytogenes* and *Y. enterocolitica.*

## Figures and Tables

**Table 1:** The number and percentage of chemical elements present in *Brevibacillus* sp. MAHUQ-41-derived AgNPs.

Element	Weight%	Atomic%
Cu K	39.02	52.07
Ag L	60.98	47.93
Totals	100.00	100.00

**Table 2:** Antibacterial impact of *Brevibacillus* sp. MAHUQ-41-mediated green-synthesized AgNPs against *L. monocytogenes* and *Y. enterocolitica*.

Pathogenic Species	ZOI (mm)	
	50 μL	100 μL
*Listeria monocytogenes* [ATCC 19114]	9.1 ± 1.0	19.1 ± 1.5
*Yersinia enterocolitica* [ATCC 9610]	9.0 ± 1.1	18.9 ± 1.3

**Table 3:** Antimicrobial impact of tested antibiotics against *L. monocytogenes* and *Y. enterocolitica*. -, No ZOI.

Pathogenic Species	Antibiotic	ZOI (mm)
*Listeria monocytogenes* [ATCC 19114]	Lincomycin	-
	Penicillin G	-
	Novobiocin	-
	Oleandomycin	-
	Vancomycin	8.9 ± 1.2
	Erythromycin	-
*Yersinia enterocolitica* [ATCC 9610]	Lincomycin	-
	Penicillin G	-
	Novobiocin	10.2 ± 1.0
	Oleandomycin	-
	Vancomycin	-
	Erythromycin	-

## Data Availability

The original contributions presented in this study are included in this article. Further inquiries can be directed to the corresponding author.
